# Perceived increased susceptibility to COVID-19 due to smoking was associated with reduced smoking at home but not on the streets amid the pandemic: A population-based cross-sectional study

**DOI:** 10.18332/tid/161860

**Published:** 2023-05-05

**Authors:** Ying Yao, Derek Yee Tak Cheung, Tzu Tsun Luk, Tai Hing Lam, Yongda Socrates Wu, Man Ping Wang

**Affiliations:** 1 School of Nursing, The University of Hong Kong, Hong Kong, People’s Republic of China; 2 School of Public Health, The University of Hong Kong, Hong Kong, People’s Republic of China

**Keywords:** risk perception, COVID-19, smoking, tobacco, changes

## Abstract

**INTRODUCTION:**

Perceived risk of COVID-19 infection is associated with smoking behaviors, but the change in smoking across different settings are uncertain. We examined the associations of perceived increased susceptibility to COVID-19 due to smoking with change in smoking at home and on the streets.

**METHODS:**

We analyzed data of 1120 current cigarette smokers aged ≥15 years from a population-based telephone survey in Hong Kong. Perceived increased susceptibility to COVID-19 due to smoking, change in smoking, intention to quit, and tobacco dependence were measured. We used Poisson regression with robust variance to estimate adjusted risk ratio (ARR) for associations, adjusting for sociodemographic characteristics, intention to quit, and time to first cigarette after waking.

**RESULTS:**

More current smokers reduced smoking on the streets (46.1%; 95% CI: 42.8–50.0) than at home (8.7%; 95% CI: 7.0–10.8). Perceived increased susceptibility to COVID-19 due to smoking was associated with smoking reduction at home (ARR=3.29; 95% CI: 1.80–6.00, p<0.001) but not on the streets (ARR=1.13; 95% CI: 0.98–1.30, p=0.09). More smokers with stronger quit intention and lower tobacco dependence reduced smoking at home but not on the streets in those with high perceived increased susceptibility to COVID-19 due to smoking.

**CONCLUSIONS:**

This is the first report showing that more cigarette smokers reduced smoking on the streets than at home, and the perceived increased susceptibility to COVID-19 due to smoking was only associated with smoking reduction at home but not on the streets. Improving smokers’ awareness of the susceptibility to COVID-19 may be an effective strategy to reduce tobacco consumption and secondhand smoke exposure at home within the context of future respiratory pandemics.

## INTRODUCTION

Smokers are more likely to suffer severe outcomes of COVID-19 including hospitalization, intensive care unit admission, and death^1,2^. A systematic review and meta-analysis reported patients with non-communicable diseases (such as cardiovascular diseases, chronic respiratory diseases, and cancers) had a greater risk for developing serious COVID-19 outcomes^3^, and smoking is a risk factor for the aforementioned non-communicable diseases^4^. Smoking with frequent hand and mouth contact could predispose smokers to COVID-19 from contaminated hands, cigarettes, and infected people around them^5,6^. The World Health Organization (WHO) has highlighted the importance of smoking cessation in reducing severe COVID-19 outcomes^7^.

Non-pharmaceutical interventions amid COVID-19 could affect smokers’ smoking behaviors. Specifically, social distancing and working from home could help smokers avoid their usual triggers and provide an opportunity to break the habit^8^. Conversely, the absence of environmental restrictions (e.g. smoke-free workplaces) may have led to an increase in daily smoking^8^. Changes in smoking during COVID-19 differ across countries. Within the same population, some smokers decreased smoking, while others increased or maintained consumption^9-14^. In Hong Kong, we found that 23.1% of 1595 adult smokers reduced smoking (including quitting), 11.6% increased smoking and 65.3% did not change at the early stage of the pandemic when restrictions on social gatherings and mandatory wearing of masks (29 July 2020) were implemented by the government^15^. Changes in smoking may be different at home and on the streets amid COVID-19 due to varied non-pharmaceutical interventions. Smoking at home could increase as people spent most of their time at home due to the work-from-home policy and home isolation measures. This could lead to a substantial increase in secondhand smoke exposure at home in family members. Smoking on the streets could be reduced due to social distancing and mask restrictions. Identifying the changes in smoking at home and on the streets separately amid the COVID-19 pandemic can help to formulate tobacco control measures tailored for different venues (such as penalty for mask off while smoking). To the best of our knowledge, no data which differentiated the changes in smoking at home and on the streets are available in the literature.

The perceived risk of COVID-19 infection could increase people’s proactive prevention behaviors, such as wearing masks and washing hands^16,17^. Studies from the UK showed that smokers were more concerned about contracting COVID-19 than never smokers^11,18^. Perceived risk of COVID-19 infection appears to be associated with increased motivation to quitting^19,20^. In Hong Kong, 43% of 201 youth smokers (aged <25 years) who used our quitline service reported that the pandemic increased their intention to quit smoking^21^. We also found in 659 adult smokers that perceived susceptibility to COVID-19 infection was associated with smoking reduction^22^. Examining how perceived increased susceptibility to COVID-19 due to smoking affects changes in smoking at home and on the streets could inform tobacco control policies amid the COVID-19 pandemic and help governments and cessation services allocate resources accordingly. To date, little is known about the associations of perceived increased susceptibility to COVID-19 due to smoking with changes in smoking at home and on the streets. We examined these associations using data from our population-based telephone surveys. We also explored if intention to quit and tobacco dependence were effect modifiers given their associations with smoking behaviors. Specifically, we hypothesized that more smokers reduced smoking on the streets and perceived increased susceptibility to COVID-19 due to smoking was associated with reduced smoking both at home and on the street.

## METHODS

### Study design

Data were from the 2019 Tobacco Control Policy-related Survey (TCPS) of the Hong Kong Council on Smoking and Health with the consultancy of the Schools of Nursing and Public Health of the University of Hong Kong. Verbal informed consent was obtained before interviews.

**Figure 1:**
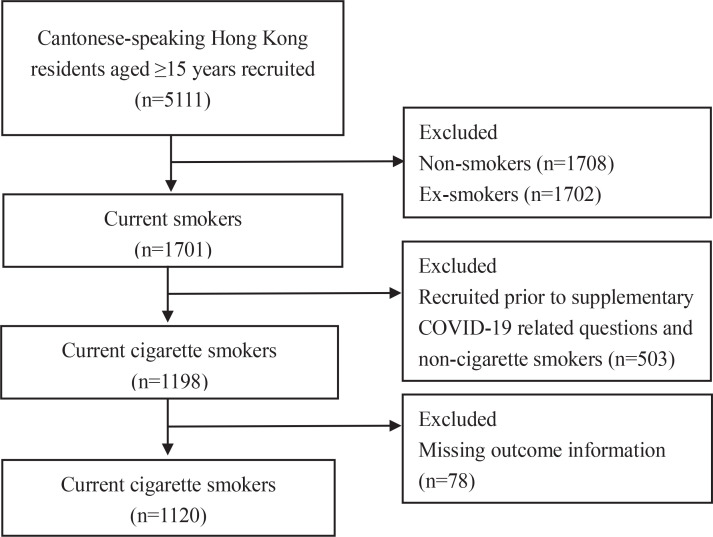
Flow diagram of numbers of included and excluded respondents

The survey fieldwork was conducted from December 2019 to October 2020 in Hong Kong. The methods and procedures were similar to the previous TCPS^23^. Briefly, a population-based telephone survey was carried out by the Public Opinion Research Institute, one of the well-known local survey agencies. Cantonese-speaking Hong Kong residents aged ≥15 years (n=5111) were recruited randomly from a telephone list which was generated by using the ‘plus/minus one/two’ method on a residential telephone directory. Computer-assisted telephone interviews (CATI) using an anonymous and structured questionnaire were conducted on eligible respondents (one selected from a household by the ‘next birthday’ approach). Three target groups were included: current smokers (n=1701) who smoked (including any forms of tobacco products) either daily or occasionally (e.g. non-daily, once every few weeks), ex-smokers (n=1702) who smoked in the past but had stopped, and never smokers (n=1708)^24^. Smokers and ex-smokers were over-sampled to improve the precision of the estimate given that the relatively small proportion of daily cigarette smokers (10.2% in 2019) in Hong Kong. Questions on COVID-19 were later added and asked in current smokers recruited from 6 July 2020 to 15 October 2022, amid the third wave of COVID-19 outbreak in Hong Kong. On 29 July 2020, the mandatory wearing of masks in outdoor places was implemented, but only 7 respondents had been recruited, therefore we did not conduct subgroup analyses. Since only cigarette users provided information on the intention to quit and tobacco dependence, only current cigarette smokers (n=1120) were included in the analysis (Figure 1).

### Measurements

Change in smoking at home during COVID-19 was ascertained by asking: ‘Did you change your tobacco (all products) consumption at home amid the COVID-19 pandemic?’. There were 10 response options: ‘no smoking at home before and amid the outbreak’, ‘only smoking at home before the outbreak’, ‘only smoking at home amid the outbreak’, ‘reduced a lot’, ‘reduced a bit’, ‘no change’, ‘increased a bit’, ‘increased a lot’, ‘do not know/remember’, and ‘refuse to answer’. Change in smoking on the streets was asked in the same manner. These responses were combined into 3 groups of ‘reduced’, ‘unchanged’ and ‘increased’ for data analysis. Respondents who answered ‘do not know/remember’ or ‘refused to answer’ (n=12) were excluded from all analyses. Current smokers who did not smoke at home (n=69) or on the streets (n=137) were also excluded from the corresponding analysis. Increases in smoking at home (n=67) and on the streets (n=10) were uncommon, and therefore were combined with ‘unchanged’ in the analysis.

Perceived increased susceptibility to COVID-19 due to smoking was ascertained by asking: ‘Do you think smoking tobacco products (e.g. conventional cigarettes, e-cigarettes, heated tobacco products, or other alternative tobacco products) will increase the chance of contracting COVID-19?’. The responses were on a scale from 0 (do not increase the chance of infection), 5 (half and half), to 10 (very likely to increase the chance of infection). Based on the median (4), the responses were dichotomized into ‘median or below (≤4)’ and ‘above median (>4)’ for data analysis.

Tobacco dependence was assessed by the minutes to the first cigarette after waking with 4 response options: ≤5, 6–30, 31–60, and >60 minutes^25^. The later from waking up the first cigarette is, the weaker the tobacco dependence. Intention to quit was assessed by respondents’ planned quit date with 7 response options: ‘today’, ‘in 7 days’, ‘in 30 days’, ‘in 6 months’, ‘after 6 months’, ‘undecided’, and ‘no intention’^26^. These options were combined and analyzed as ‘within 6 months’, ‘after 6 months/undecided’, and ‘no intention’. The earlier the planned to quit date, the stronger the quit intention. Sex, age, education level, employment status, monthly household income, living together with children, and the self-rated health of respondents were recorded. Being an employer, employee or self-employed was classified as economically active; and being a student, housekeeper, retired or unemployed was classified as economically inactive.

### Statistical analysis

Stata version 15.1 (StataCrop LLC, College Station, TX, USA) was used for data analysis. All descriptive data were weighted according to the sex and age distribution of current smokers in Hong Kong in 2019^24^ to improve the representativeness of the sample. Chi-squared tests and Wilcoxon rank-test were used to compare changes in smoking at home and on the streets across sociodemographic status, perceived increased susceptibility to COVID-19, intention to quit, and tobacco dependence. Poisson regression with robust variance was used to estimate the adjusted risk ratio (ARR)^27^ for changes in smoking in relation to the perceived increased susceptibility to COVID-19 due to smoking, adjusting for sex, age, education level, employment status, household income, living together with children, self-rated health status, intention to quit, and time to first cigarette after waking. Average marginal effects (AMEs) were used to measure risk differences among respondents with different levels of characteristics. Multiple imputation by chained equation was used to impute missing data, under the assumption that data were missing at random^28^. The imputation models included all variables in the analytical models. Estimates were inferred from 40 sets of imputed data. Targeted maximum likelihood estimation (TMLE) was also conducted as a sensitivity analysis for results from multiple Poisson models. This method improves the chance of correct model specification by: 1) only requiring correct specification of either the exposure or the outcome model; and 2) nonparametric machine-learning methods employed in model specification. A two-sided p<0.05 was considered statistically significant.

## RESULTS

Among 1120 current cigarette smokers, 918 (77.7%) smoked cigarettes only, 23 (2.9%) smoked cigarettes and heated tobacco, 23 (2.7%) smoked cigarettes and e-cigarette, 64 (6.3%) smoked cigarettes and cigar, and 64 (7.6%) smoked two or more other tobacco products in addition to cigarettes (Supplementary file Table 1). Supplementary file Table 2 shows that 8.7% of 1120 cigarette smokers (95% CI: 7.0–10.8) reduced smoking at home, while 46.1% (95% CI: 42.8–49.5) reduced smoking on the streets. However, 7.5% (95% CI: 5.8–9.6) of smokers increased smoking at home, and only 1.1% (95% CI: 0.6–2.2) increased smoking on the streets. Table 1 shows that 83.6% of cigarette smokers were male, 27.2% were aged ≥60 years, and 58.4% had secondary education. Near two-thirds (64.3%) were economically active and 19.4% had a monthly household income of HK$60000 or above (HK$7.8=US$1). About one-fifth (22.1%) were living together with children, and 35.2% perceived their health as average.

**Table 1:** Sociodemographic characteristics of current cigarette smokers, by change in smoking at home and on the streets amid COVID-19, Hong Kong, 2020 (N=1120)

*Characteristics*	*Total*	*Change in smoking at home*			*Change in smoking on the streets*		
	*(N=1120) %*	*Unchanged/increased (N=958) %*	*Reduced (N=92) %*	*p*	*Unchanged / increased (N=500) %*	*Reduced (N=471) %*	*p*
**Sex**				0.53			0.34
Male	83.6	90.9	9.1		49.6	50.4	
Female	16.4	89.2	10.8		44.8	55.2	
**Age** (years)				0.54			0.08
15–29	6.7	88.0	12.0		39.2	60.8	
30–39	18.2	92.2	7.8		43.5	56.5	
40–49	22.7	88.6	11.4		46.6	53.4	
50–59	25.3	89.7	10.3		49.7	50.3	
≥60	27.2	92.8	7.2		56.7	43.3	
**Education level**				0.69			0.12
Primary or lower	12.8	88.7	11.3		58.4	41.6	
Secondary	58.4	91.2	8.8		47.3	52.7	
Tertiary	28.8	90.4	9.7		48.0	52.0	
**Employment status^a^**				**0.008**			0.56
Economically active	64.3	88.8	11.2		48.2	51.8	
Economically inactive	35.7	93.7	6.3		50.3	49.7	
**Monthly household income** (HK$)				0.42			**0.02**
≤10000	16.8	92.7	7.3		57.7	42.3	
10000–19999	16.1	92.1	7.9		55.7	44.3	
20000–29999	17.7	93.8	6.2		40.3	59.7	
30000–39999	15.5	88.7	11.3		40.4	59.6	
40000–59999	14.4	87.2	12.8		40.5	59.5	
≥60000	19.4	88.6	11.4		49.9	50.1	
**Living together with children**	22.1	91.4	8.6	0.25	49.2	50.8	0.49
**Self-rated health status**				0.46			0.25
Excellent	8.1	94.7	5.3		45.9	54.1	
Very good	20.6	87.8	12.2		49.9	50.1	
Good	30.8	91.2	8.8		49.6	50.4	
Average	35.2	91.2	8.8		46.0	54.0	
Poor	5.2	87.8	12.2		39.8	60.2	

a Being an employer, employee or self-employed was classified as economically active; being a student, housekeeper, retired or unemployed was classified as economically inactive. Results were weighted by the sex and age distribution of current smokers in Hong Kong in 2019. The p-values were from chi-squared tests. HK$7.8=US$1.

Table 2 shows that smokers who reduced smoking at home had higher perceived increased susceptibility to COVID-19 due to smoking (median=5, IQR: 5–7) than those who increased smoking or remained unchanged (median=3, IQR: 0–5, p<0.001). Similarly, smokers who reduced smoking on the streets had higher perceived increased susceptibility to COVID-19 due to smoking (median=5, IQR: 0–5) than those who increased or remained unchanged (median=3, IQR: 0–5, p=0.005). More smokers with stronger intention to quit (with 6 months 19.6% vs after 6 months or undecided 10.2% vs no intention 6.3%, p<0.001) and lower tobacco dependence (after 60 minutes 13.2% vs 31–60 minutes 11.7% vs 6–30 minutes 8.3% vs within 5 minutes 3.5%, p=0.006) reported reduced smoking at home, but no associations were found for smoking on the streets.

**Table 2:** Smoking and quitting-related characteristics of current cigarette smokers, by change in smoking at home and on the streets amid COVID-19, Hong Kong, 2020 (N=1120)

*Characteristics*	*Total*	*Change in smoking at home*			*Change in smoking on the streets*		
	*(N=1120) %*	*Unchanged/increased (N=958) %*	*Reduced (N=92) %*	*p*	*Unchanged / increased (N=500) %*	*Reduced (N=471) %*	*p*
**Perceived increased susceptibility to COVID-19 due to smoking,** median (IQR)^a^	4 (0–5)	3 (0–5)	5 (5–7)	**<0.001**	3 (0–5)	5 (0–5)	**0.005**
Low (≤4)	50.0	96.0	4.0	**<0.001**	52.0	48.0	**0.03**
High (>4)	50.0	84.6	15.4		43.5	56.5	
**Intention to quit**				**<0.001**			0.42
No intention	56.2	93.7	6.3		50.0	50.0	
After 6 months/undecided	31.0	89.8	10.2		49.4	50.6	
Within 6 months	12.8	80.4	19.6		42.5	57.5	
**Minutes to first cigarette after waking**				**0.006**			0.79
≤5	22.7	96.5	3.5		46.2	53.8	
6–30	28.3	91.7	8.3		45.2	54.8	
31–60	12.8	88.3	11.7		46.1	53.9	
>60	36.1	86.8	13.2		49.7	50.3	

a Perceived increased susceptibility to COVID-19 due to smoking scored from 0 to 10 (median=4); and classified into two groups low (≤4) and high (>4) based on the median with a higher score indicating higher susceptibility to COVID-19 due to smoking. Results were weighted by the sex and age distribution of current smokers in Hong Kong in 2019. The p-values were from chi-squared tests, and median from Wilcoxon rank-test. IQR: interquartile range.

Table 3 shows that higher perceived increased susceptibility to COVID-19 due to smoking was only associated with reduced smoking at home (ARR=3.29; 95% CI: 1.80–6.00, p<0.001) but not on the streets (ARR=1.13; 95% CI: 0.98–1.30, p=0.09). TMLE showed the consistent results [RR=3.31; 95% CI: 1.71–6.43 (at home) and RR=1.14; 95% CI: 0.99–1.32 (on the streets)]. Intention to quit (within 6 months, ARR=2.67; 95% CI: 1.46–4.88, p<0.01) and time to first cigarette after waking (in 31–60 minutes, ARR=2.92; 95% CI: 1.20–7.11, p<0.05; after 60 minutes, ARR=2.39; 95% CI: 1.03–5.57, p<0.05) were associated with reduced smoking at home but not on the streets.

**Table 3:** The association of reduced smoking of current cigarette smokers, at home and on the streets amid COVID-19, with perceived increased susceptibility to COVID-19 due to smoking, intention to quit and tobacco dependence, Hong Kong, 2020 (N=1120)

	*Reduced (vs unchanged/increased) smoking at home ARR (95% CI)*			*Reduced (vs unchanged/increased) smoking on the streets ARR (95% CI)*		
	*Adjusted^a^*	*Imputed^b^*	*Sensitivity analysis by TMLE^c^*	*Adjusted^a^*	*Imputed^b^*	*Sensitivity analysis by TMLE^c^*
**Perceived increased susceptibility to COVID-19 from smoking** (score: 0–10, median=4)						
Low (≤4) (Ref.)	1	1	1	1	1	1
High (>4)	3.29 (1.80–6.00)***	3.12 (1.82–5.38)***	3.31 (1.71–6.43)	1.13 (0.98–1.30)	1.16 (1.01–1.34)*	1.14 (0.99–1.32)
**Perceived increased susceptibility to COVID-19 from smoking** (score: 0–10, continuous)	1.23 (1.14–1.33)***	1.23 (1.15–1.31)***	-	1.02 (1.00–1.05)	1.03 (1.00–1.05)*	-
**Intention to quit**						
No intention (Ref.)	1	1	-	1	1	-
After 6 months/undecided	1.36 (0.76–2.45)	1.74 (1.07–2.83)*	-	1.00 (0.85–1.17)	1.02 (0.88–1.19)	-
Within 6 months	2.67 (1.46–4.88)**	2.76 (1.64–4.65)***	-	1.13 (0.92–1.39)	1.10 (0.90–1.35)	-
Intention to quit (continuous)	1.87 (1.42–2.47)***	1.98 (1.55–2.55)***	-	1.07 (0.97–1.18)	1.05 (0.95–1.15)	
**Minutes to first cigarette after waking**						
≤5 (Ref.)	1	1	-	1	1	-
6–30	1.59 (0.66–3.83)	2.15 (1.00–4.64)	-	0.96 (0.81–1.15)	0.99 (0.83–1.19)	-
31–60	2.92 (1.20–7.11)*	2.75 (1.17–6.47)*	-	0.92 (0.72–1.16)	0.96 (0.75–1.21)	-
>60	2.39 (1.03–5.57)*	2.71 (1.28–5.00)**	-	0.80 (0.66–0.97)	0.88 (0.73–1.06)	-
**Time to first cigarette after waking (continuous)**	1.37 (1.14–1.64)***	1.49 (1.44–1.54)***	-	0.95 (0.90–1.01)	0.97 (0.92–1.03)	-

a Sex, age, education level, employment status, monthly household income, having children living together, self-rated health status, intention to quit, and time to first cigarette after waking were mutually adjusted.

b Analysis based on imputed data. Sex, age, education level, employment status, monthly household income, having children living together, self-rated health status, intention to quit, and time to first cigarette after waking were mutually adjusted.

c The casual risk ratios for the associations of change in smoking at home and on streets amid COVID-19 with perceived increased susceptibility to COVID-19 from Targeted Maximum Likelihood Estimation (TMLE); TMLE improves the chance of correct model specification by: 1) only requiring correct specification of either the exposure or the outcome model; 2) nonparametric machine-learning methods employed in model specification. ARR: adjusted risk ratio.

* p<0.05;

** p<0.01;

*** p<0.001.

Supplementary file Table 3 shows the risk difference of reducing smoking at home/on the streets in relation to perceived increased susceptibility to COVID-19 due to smoking. A high perceived increased susceptibility to COVID-19 due to smoking was associated with a larger risk in smokers with stronger quit intention (within 6 months 18.6% vs after 6 months/undecided 9.8% vs no intention 6.7%, p=0.02) and with lower tobacco dependence who had reduced smoking at home (after 60 minutes 12.1% vs 31–60 minutes 12.8% vs 6–30 minutes 7.4% vs within 5 minutes 4.6%, p=0.03). However, no significant differences were found for reducing smoking on the streets (intention to quit, p=0.63; time to the first cigarette after waking, p=0.65).

## DISCUSSION

This is the first report on the associations of perceived increased susceptibility to COVID-19 due to smoking with change in smoking at home and on the streets. In this population sample of current cigarette smokers in Hong Kong, nearly half (46.1%) of smokers reduced smoking on the streets, but only 8.7% did so at home. High perceived increased susceptibility to COVID-19 due to smoking was only associated with reduced smoking at home but not on the streets. The robustness of the finding was corroborated by the sensitivity analysis (TMLE).

More smokers reported an increase in smoking at home, which can be explained by prolonged stays at home and emotional distress amid COVID-19, as increased smoking is often used as a strategy to cope with stress, anxiety, and boredom amid the pandemic^29,30^. Conversely, more smokers reduced smoking on the streets than at home, which can be attributed to the inconvenience of smoking on the streets due to the restrictions on going out amid the pandemic. Our previous observational study in Hong Kong also showed that the volume of smokers decreased significantly in outdoor smoking hotspots (the most common public places where smokers gather to smoke) since the outbreak of COVID-19^31^. Almost 100% voluntary masking was observed for about 6 months before the government implemented mandatory masking in outdoor places from 29 July 2020 in Hong Kong^32^. As smoking was explicitly stated as being not exempted, this could explain the reduction in outdoor smoking as a response to regulations. Future studies are warranted to clarify whether smoking in outdoor places will resume after the pandemic ends or the related regulations are lifted.

Consistent with previous reports, the perceived probability of COVID-19 infection was associated with smoking reduction^18,19^. We further found current smokers with high perceived increased susceptibility to COVID-19 due to smoking were associated with 2.29 times increased likelihood of reduced smoking at home. Our previous community-based cross-sectional survey reported that perceived increased susceptibility to COVID-19 due to smoking was associated with a 75% increase in the likelihood of smoking reduction (overall smoking reduction, no location distinction)^22^. Reducing smoking at home amid the pandemic is important for households reducing secondhand smoke exposure, as family members were staying at home longer than ever amid COVID-19^33,34^. Hence, improving smokers’ awareness of the susceptibility to COVID-19 may be an effective strategy to reduce tobacco consumption and secondhand smoke exposure at home in the context of COVID-19 and beyond.

However, we found that high perceived increased susceptibility to COVID-19 due to smoking was not associated with reduced smoking on the streets. This might suggest that reduced tobacco consumption on the streets was probably due to COVID-19 preventive measures (such as masking mandatory and social distancing) rather than individual intrinsic motivation. Strong enforcement with heavy penalties for smokers who take off their masks to smoke in public places to curb smoking on the streets during the pandemic is warranted.

Stronger intention to quit and lower tobacco dependence were only associated with reducing smoking at home in smokers who perceived increased susceptibility to COVID-19 due to smoking. Individual intrinsic motivation and self-efficacy, social normative, and cultural environment are the main factors in smoking motivation^35^. In homes that are not restricted by tobacco control policies, smoking reductions are mostly dependent on smokers’ smoking intention. Hence, interventions on improving smokers’ intrinsic motivation to quit and self-efficacy to refrain from smoking at home would be urgently needed when the stringent pandemic control measures are relaxed or dropped in the future.

### Limitations

Several limitations of our study should be noted. First, causal inference could not be drawn due to the cross-sectional design. Second, recall bias and social desirability bias might exist in our self-reported data. Third, ex-smokers who had quit after the onset of COVID-19 were not included, which might lead to underestimation of the association if reduced smoking was due to the perceived increased susceptibility to COVID-19 infection, but we had asked ex-smokers if they stopped smoking after the outbreak, and few did so (n=7, excluded). Fourth, changes in smoking could be affected by non-pharmaceutical interventions, and our findings may not be applicable to other countries/regions with different prevention policies. Fifth, even though we adjusted for sociodemographic factors and known predictors of smoking cessation (including the intention to quit and time to the first cigarette after waking)^36^, unmeasured or residual confounding could not be excluded (such as readiness to quit before COVID-19), but the result of TMLE indicated that our main findings should be reliable.

## CONCLUSIONS

This is the first report showing that more cigarette smokers reduced smoking on the streets than at home, and the perceived increased susceptibility to COVID-19 due to smoking was only associated with smoking reduction at home but not on the streets. Improving smokers’ awareness of the susceptibility to COVID-19 may be an effective strategy to reduce tobacco consumption and secondhand smoke exposure at home in the context of COVID-19 and beyond.

## Data Availability

The data supporting this research are available from the authors on reasonable request.
