# Supplementary material for: Perceived increased susceptibility to COVID-19 due to smoking was associated with reduced smoking at home but not on the streets amid the pandemic: A population-based cross-sectional study
Source: Tob Induc Dis. 2023 May 5;21:56. doi: 10.18332/tid/161860 (PMC10161687; doi:10.18332/tid/161860)
Supplement: Supplementary file 1 [file TID-21-56-s1.pdf]

Supplementary table 1. Co-use of other tobacco products by current cigarette smokers in Hong Kong, 2020 (N=1120 current cigarette smokers)

Tobacco products	n	Crude %	Weighted %
Cigarette only	918	81.9	77.7
Cigarette and heated tobacco	23	2.1	2.9
Cigarette and e-cigarette	23	2.1	2.7
Cigarette and waterpipe	12	1.1	1.3
Cigarette and cigar	64	5.7	6.3
Cigarette and hand-rolled cigarettes	8	0.7	0.8
Cigarette and pipe	7	0.6	0.6
Cigarette and snuff	1	0.1	0.1
Cigarette and two or more other products	64	5.7	7.6
Total	1120	100.0	100.0

Results were weighted by the sex and age distribution of current smokers in Hong Kong in 2019.[25]

Supplementary table 2. Change in smoking at home and on the streets amid COVID-19 in Hong Kong, 2020 (N=1120 current cigarette smokers)

	Crude n (%)	Weighted % (95% CI)
Change in smoking at home		
Increased	67 (6.0)	7.5 (5.8,9.6)
Unchanged	891 (79.5)	76.6 (73.5,79.5)
Reduced	92 (8.2)	8.7 (7.0,10.8)
Not smoking at home before and amid the outbreak	69 (6.2)	7.2 (5.5,9.3)
DK/RTA	1 (0.1)	0.0 (0.0,0.3)
Total	1120 (100.0)	100.0
Change in smoking on the streets		
Increased	10 (0.9)	1.1 (0.6,2.2)
Unchanged	490 (43.8)	42.9 (39.7,46.3)
Reduced	471 (42.1)	46.1 (42.8,49.5)
Not smoking on the streets before and amid the outbreak	137 (12.2)	9.0 (7.5,10.8)
DK/RTA	12 (1.1)	0.8 (0.4,1.6)
Total	1120 (100.0)	100.0

DK/RTA: Don't know /refused to answer.
Results were weighted by the sex and age distribution of current smokers in Hong Kong in 2019.[25]
CI: confidence interval.

Supplementary table 3. Risk difference (average marginal effects) of reducing smoking at home/on the streets in relation to perceived increased susceptibility to COVID-19 due to smoking (N=1120 current cigarette smokers)

	Risk difference for high (vs. low) perceived increased susceptibility to COVID-19 due to smoking [a]	
	Reduced (vs. unchanged or increased) smoking at home % (95% CI)	Reduced (vs. unchanged or increased) smoking on the streets % (95% CI)
Intention to quit		
No intention	6.7 (2.8,10.6) **	6.4 (-1.0,13.8)
After 6 months / Undecided	9.8 (4.8,14.7) ***	6.6 (-0.8,14.0)
Within 6 months	18.6 (8.6,28.6) ***	7.2 (-0.9,15.2)
P-value for overall comparison	0.02	0.63
Time to first cigarette after waking		
Within 5 minutes	4.6 (0.4,8.7) *	7.1 (-1.1,15.3)
In 6 – 30 minutes	7.4 (3.3,11.5) ***	6.9 (-1.0,14.9)
In 31 – 60 minutes	12.8 (5.0,20.7) **	6.4 (-0.9,13.8)
After 60 minutes	12.1 (5.8,18.4) ***	5.9 (-0.8,12.6)
P-value for overall comparison	0.03	0.65

Based on the adjusted model in Table 3, i.e., sociodemographic characteristics, perceived increased susceptibility to COVID-19 due to smoking, intention to quit and time to first cigarette after waking were mutually adjusted.
[a] Perceived increased susceptibility to COVID-19 due to smoking scored from 0 to 10. It was classified into two groups "low (≤4)" and "high (>4)" based on the median (4), with "low" group as the reference.
* P<0.05; ** P<0.01; *** P<0.001.
CI: confidence interval.

Supplementary table 4. Summary of missing observations (> 5%) for related variables

Variables	Missing observation (%)
Monthly household income	145 (14.9)
Change in smoking on the streets	149 (15.3)
Perceived increased susceptibility to COVID-19 due to smoking	133 (13.5)
Change in smoking at home	70 (6.7)
Time to first cigarette after waking	65 (6.2)

© 2023 Yao Y. et al.
